# Occult Breast Cancer Presenting as Leptomeningeal Carcinomatosis

**DOI:** 10.4021/wjon408w

**Published:** 2012-04-23

**Authors:** Megan E. McNally, William Carson

**Affiliations:** a Division of Surgical Oncology, Department of Surgery, The Ohio State University, USA

**Keywords:** Leptomeningeal carcinomatosis, Palliation

## Abstract

Leptomeningeal carcinomatosis (LC) is a rare and devastating metastatic manifestation of both liquid and solid tumors consisting of dissemination of malignant cells with invasion into the meninges. Few options exist in most clinical situations, especially when LC is the presenting sign of occult malignancy. The prognosis is often poor with limited survival. Aims of palliation must be considered the primary goal for most patients. We report a case in which occult metastatic breast cancer presented with neurological symptoms from LC. We discuss diagnosing the primary malignancy when LC is the presenting manifestation as well as treatment, both palliative and cytoreductive. We also focus on those patients with breast cancer that are at highest risk of developing LC.

## Introduction

Leptomeningeal carcinomatosis (LC) is a rare and devastating metastatic manifestation of both liquid and solid tumors consisting of dissemination of malignant cells with invasion into the meninges. Rarely is it the presenting symptom of malignancy; however, when it is, it can be difficult to define the primary tumor of origin and survival is limited [1].

## Case Report

**Figure 1:**
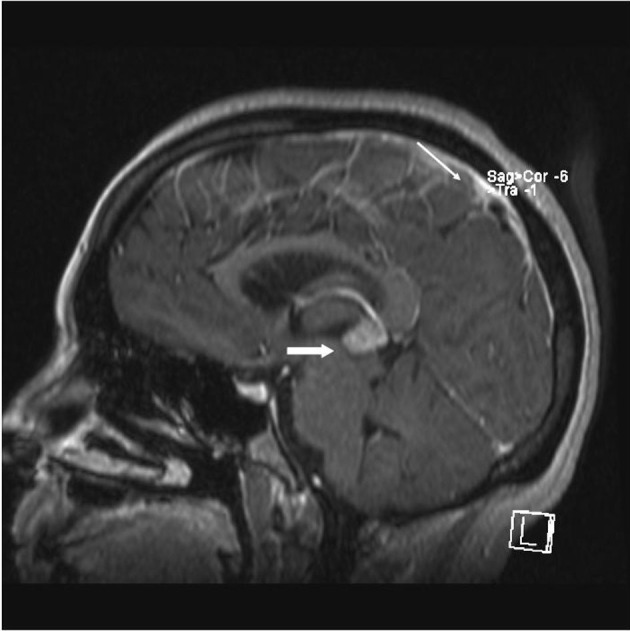
MRI brain (saggital) reveals obstructive hydrocephalus caused by an enhancing pineal gland mass (large arrow) and leptomeningeal carcinomatosis (LC) (small arrow).

**Figure 2:**
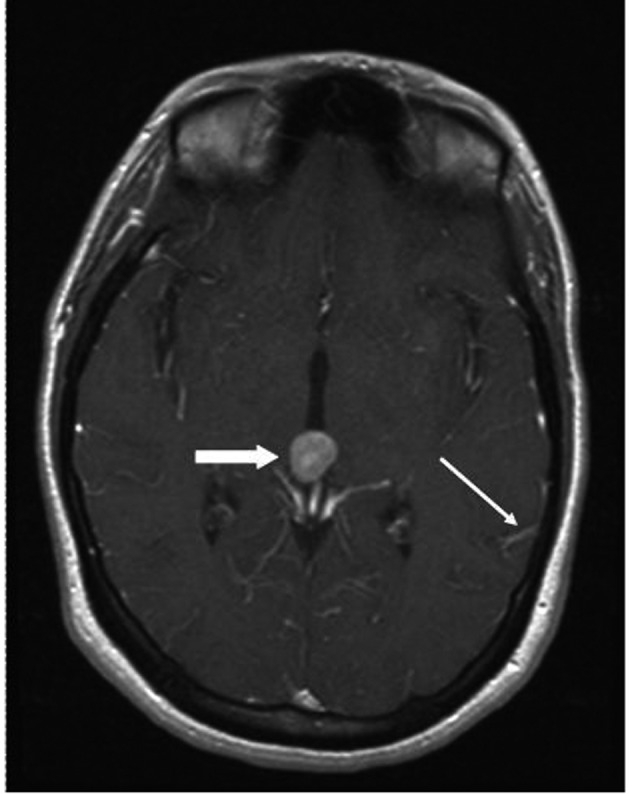
MRI brain (transversal) reveals obstructive hydrocephalus caused by an enhancing pineal gland mass (large arrow) and leptomeningeal carcinomatosis (LC) (small arrow).

A 33 year old woman presented to the Emergency Department with bifrontal headaches and blurry vision. Her past medical history was significant for Crohn’s disease which required a subtotal colectomy; she was not on any medications at presentation. Physical examination was without additional abnormalities. Magnetic resonance imaging (MRI) of the head revealed an enhancing pineal gland mass (Fig.1 and 2, large arrow) and obstructive hydrocephalus. These findings were concerning for either a primary or secondary brain malignancy. Cerebral spinal fluid (CSF) examination revealed malignant cells from an unknown primary.

The patient was admitted, and a ventriculostomy was placed to treat the hydrocephalus. When the patient stabilized, she received whole brain external beam radiation (3000 cGy in 10 fractions), which resulted in improvement of her vision. MRI defined the pineal gland metastasis and LC as the cause for the hydrocephalus (Fig. 1 and 2, small arrow). The LC progressed despite radiation necessitating the conversion of the ventriculostomy to a ventriculo-peritoneal shunt for the persistent symptomatic hydrocephalus.

Work up to define the primary malignancy included physical exam, upper and lower endoscopy, lab studies, chest x-ray, CT of the abdomen and pelvis, and CSF cytology. A spinal MRI to evaluate the degree of LC noted concerning areas in the breasts. Diffuse activity in both breasts was noted on CT/PET. A breast MRI and ultrasound confirmed the presence of bilateral breast masses suspicious for multi-centric breast cancer. These were not appreciable on initial physical examination due to severe fibrocystic disease. An ultrasound-guided biopsy confirmed the diagnosis of breast cancer. The final pathology was consistent with an invasive carcinoma, favoring mammary ductal type with areas suspicious for lymphovascular invasion. The tumor was triple receptor (estrogen, progesterone, HER2/neu) negative.

After multidisciplinary discussion, it was decided that intrathecal cytotoxic chemotherapy would not be beneficial due to extensive LC and persistent obstructive hydrocephalus. Instead, high dose intravenous methotrexate was initiated to treat the patient’s neurologic metastases. She tolerated two treatments at a dose of 3.5 g/m^2^. Pain secondary to cauda equina syndrome was managed with oral narcotics. Focused radiotherapy was initiated to the bony metastases but poorly tolerated. Despite whole brain irradiation and chemotherapy, progression of her LC and enlargement of the pineal gland metastasis occurred. This patient’s overall survival was three months.

## Discussion

Solid tumors such as breast cancer, lung cancer, and melanoma as well as leukemias and lymphomas can progress to leptomeningeal carcinomatosis (LC)-also referred to as carcinomatosis meningitis or neoplastic meningitis. A rare and devastating disease, LC consists of CSF dissemination of malignancy with invasion into the meninges [1]. Up to 20% of cancer patients with neurological symptoms will have meningeal disease on autopsy [2, 3].

Defining the primary tumor in a patient presenting with LC rarely affects the overall outcome; although, an exhaustive search should be performed as it may better define systemic chemotherapy options. The CNS disease should be managed early without delay regardless if a primary tumor is identified [4, 5]. The work up of the primary tumor in a patient with LC includes specific CSF cytopathologic investigations (immunohistochemistry, electron microscopy, molecular diagnosis) and modern imaging technology (computed tomography (CT), Positron Emission Tomography (PET) scan and MRI).

In breast cancer, CNS metastases are the fourth most common distant metastatic site after the bone, lungs, and liver [6]. Among solid tumors, breast cancer invades the meninges most frequently [7]. Anywhere from 5 - 50% of patients with metastatic breast cancer will also have CNS metastases, of which up to 15% will be occult [8, 9]. Specifically, 2 - 5% of patients with breast cancer ultimately will develop LC [10]. CNS disease typically appears within two-three years after the diagnosis of metastatic disease [9-12]. It is common for most patients to have intraparenchymal brain metastases concurrent with LC [3, 7]. The overall incidence of LC appears to be increasing but this may be related to several factors including more sensitive neurological imaging, improved overall survival, and increased awareness by clinicians [11, 12].

As systemic therapies and overall survival improve, likely more patients with metastatic breast cancer will develop LC. Patients at high risk include those with HER2/neu expressing tumors, especially those treated with trastuzumab (Herceptin™) compared to traditional chemotherapy [7]. This phenomenon is thought to be due to three factors: predilection of HER2/neu expressing tumors for spread to the CNS; poor penetration of trastuzumab through the blood brain barrier; and improved visceral disease control with overall longer survival allowing for late CNS spread [6]. Other risk factors include estrogen receptor negative tumors [13] and younger patients with aggressive tumors. The median age of these patients is typically five years younger than that of patients who present with metastatic disease but without CNS involvement [14, 15].

The presentation of LC is due to symptoms from the obstruction of normal CSF flow, local tumor infiltration, alteration in the metabolism of the CNS, or a combination of these factors [12]. Only 5 - 10% of patients will present with CNS disease as the first manifestation of cancer [14, 15]. These symptoms include focal neurologic deficits, pain and radiculopathies from local infiltration into the brain or spinal cord, seizures, encephalopathy, hydrocephalus, and infarcts related to occlusion of intracranial blood vessels. Typically, neurologic imaging and CSF examination will make the diagnosis [3]. The reported sensitivity for contrast enhanced MRI is 60 - 75% for LC [16, 17]. CSF findings may include a low glucose and positive cytology-an absolute criterion for diagnosis. The sensitivity of CSF cytology approaches 98% when three or more separate samples are examined [18]. Tumor markers in CSF specimens can aid in the identification of the primary tumor. CSF flow studies are helpful in those patients who are asymptomatic and in whom radiographic imaging is unclear. These flow studies may also be helpful in determining if intrathecal therapy is a feasible therapeutic option [19-21]. However, because of the overall poor sensitivity and specificity, CSF studies make for a poor evaluator of response to therapy. In patients whose radiographic and CSF studies are non-diganostic and there is no systemic manifestation of disease, a meningeal biopsy from an MRI-enhancing area may be diagnostic [22].

Typically, spread to the CNS is diagnosed in the last weeks to months of life in metastatic breast cancer. Without treatment, median survival is 4 - 6 weeks. With treatment, median survival is 4 - 13 months [1, 7, 23-25]. Half of breast cancer patients with CNS metastases die from the neurologic disease despite their systemic disease under reasonable control. In contrast, other solid tumor patients with CNS involvement typically succumb to systemic disease [7]. This phenomenon is thought to be due to the chemosensitive nature of breast cancer [14, 15, 26, 27]. For example, treatment of HER2/neu over-expressing tumors with antibody therapy (trastuzumab) has led to better control of systemic disease while leaving the CNS disease relatively under-treated. Because of the concern for neurologic progression with systemic stability, treatment of the CNS disease is paramount to improving overall survival.

For secondary brain malignancies, especially those due to spread of solid tumors, whole brain irradiation (WBI) is considered the standard treatment and shown to improve overall survival by weeks to months; it also can be effective treating LC [23, 28]. Combined treatment with WBI and intrathecal therapy improves clinical symptoms but not necessarily overall survival [29, 30]. If there is poor, altered, or obstructed CSF flow, intrathecal therapy will be less effective [19-21]. Intravenously delivered chemotherapy penetrates the CNS poorly due to the blood brain barrier but may be combined with intrathecal chemotherapy [1, 31]. In those patients with HER2/neu expressing tumors, there may be some benefit in intrathecal administration of trastuzumab. With systemic administration of trastuzumab, there is poor concentration of the drug in the CSF; the concentration can be improved by direct intrathecal administration [32].

There are several key prognostic indicators that are useful in determining who should be aggressively treated for LC and who should be offered best supportive care (BSC). Poor prognostic indicators include age > 55 years, lung metastases, cranial nerve involvement, CSF glucose < 325 mmol/l, CSF protein 0.51 - 1.0 g/l, and World Health Organization Performance Status > 3 [33, 34]. Positive CSF cytologic findings do not appear to affect overall prognosis or survival [35]. Other groups found to have limited benefit from LC-directed care include those with bulky subarachnoid or parenchymal CNS metastatic disease, radiotherapy-resistant interruption of CSF flow, and LC-related encephalopathy [20, 35]. The National Comprehensive Cancer Network (NCCN) stratifies patients into low and high risk groups using comparable criteria [36]. Patients defined as having poor prognostic factors benefit more from BSC than from aggressive LC-directed treatment. Care for these patients can include anti-epileptic drugs, anxiolytics, antidepressents and narcotics for pain control [37].

At present, the treatment of LC is inadequate. Leptomeningeal disease is a serious and life threatening manifestation of late metastatic breast cancer. A high index of suspicion in any cancer patient with neurologic symptoms must be maintained. As our systemic therapies continue to improve overall survival and reduce tumor burden, the ability for the CSF to act as a sanctuary for tumor cells increases. It is imperative to determine if there are newer treatment modalities that can target these tumor cells after they have crossed the blood brain barrier. In addition, defining the subset of the population that may benefit from aggressive screening and treatment will aid in the development of future clinical trials.
